# Supplementary material for: Revisiting superradiance dynamics from single diamond nanocrystals with a physically consistent model for fluorescence decay
Source: Nat Commun. 2026 Jan 5;17:58. doi: 10.1038/s41467-025-67847-6 (PMC12770545; doi:10.1038/s41467-025-67847-6)
Supplement: Supplementary file 1 — Supplementary Information [file 41467_2025_67847_MOESM1_ESM.pdf]

Supplementary Information for Revisiting superradiance dynamics from single diamond nanocrystals with a physically consistent model for fluorescence decay

Jakub J. Borkowski [1,*] Artur Czerwinski [1,†] and Piotr Kolenderski [1,‡]

[1] *Institute of Physics, Faculty of Physics, Astronomy and Informatics, Nicolaus Copernicus University in Torun, ul. Grudziadzka 5, 87–100 Torun, Poland*

Appendix A: Comparison of the equations in Models A and B

In Eq. (1) of the main text, we have identified the terms that differ from those in Eq. (4) by coloring them in red and enclosing them in brackets. Each term is labeled alphabetically. Supplementary Table 1 provides a reference to each differing term along with the correct replacement. For clarity, we refer to the framework presented in the commented paper as Model A [1, 2], while Model B denotes our solution, which is based on the theory of open quantum systems [3] and has been validated through numerical simulations [4].

Term label	Model A	Model B
A	$2J$	1
B	1	$2J$
C	$1-\left\|\frac{M}{J}\right\|^2$	$\left\|\frac{M}{J}\right\|^2$
D	$+2$	-2
E	$1-\left\|\frac{M+\frac{1}{2}}{J+\frac{1}{2}}\right\|^2$	$\left\|\frac{M+\frac{1}{2}}{J+\frac{1}{2}}\right\|^2$
F	$(J+M+1)$	$(J+M+1)(J-M+1)$
G	$(J+M)$	$(J+M)(J-M+1)$
H	$1-\left\|\frac{M}{J}\right\|^2$	$\left\|\frac{M}{J}\right\|^2$
I	$M(M+1)$	$M(M-1)$

Supplementary Table 1. The comparison of Model A and Model B. The labels, A-G, H, I, refer to the identified parts of Eq. (1), Eq. (2), Eq. (3), respectively, as analyzed in the main text. Columns show the corresponding factors for Model A and Model B.

* jborkowski@doktorant.umk.pl
† aczerwin@umk.pl
‡ kolenderski@umk.pl

At this stage, we have yet to interpret the marked changes. We assume that NV centers in individual domains are in collective Dicke-type states $|J,M,\sigma\rangle$ given as [2]:

$$|J,M,\sigma\rangle = \sqrt{\frac{(J+M)!(J-M)!}{(2J)!}} \sum_{perm} \Bigg| \underbrace{e_\sigma e_\sigma e_\sigma \cdots}_{J+M} \underbrace{g_\sigma g_\sigma g_\sigma \cdots}_{J-M} \Bigg\rangle, \tag{A1}$$

where the summation is performed over all possible permutations of the N_σ spins.

To describe spin dephasing we implement a local unitary phase flip operator:

$$\hat{s}_j^{(\sigma)z} = \left(|e_\sigma\rangle \langle e_\sigma|_j - |g_\sigma\rangle \langle g_\sigma|_j \right) /2, \tag{A2}$$

where the index j refers to a particular NV center. Then, we use $\hat{s}_j^{(\sigma)z}$ to compute a following amplitude:

$$\begin{aligned} \langle J,M,\sigma| 2\hat{s}_j^{(\sigma)z} |J,M,\sigma\rangle &= \frac{(J+M)!(J-M)!}{(2J)!} \\ \times \left(\sum_{perm} \Bigg\langle \underbrace{e_\sigma e_\sigma \cdots}_{J+M} \underbrace{g_\sigma g_\sigma \cdots}_{J-M} \Bigg| \right) & \left(|e_\sigma\rangle_j \otimes \sum_{perm} \Bigg| \underbrace{e_\sigma e_\sigma \cdots}_{J+M-1} \underbrace{g_\sigma g_\sigma \cdots}_{J-M} \Bigg\rangle - |g_\sigma\rangle_j \otimes \sum_{perm} \Bigg| \underbrace{e_\sigma e_\sigma \cdots}_{J+M} \underbrace{g_\sigma g_\sigma \cdots}_{J-M-1} \Bigg\rangle \right) \\ = \frac{(J+M)!(J-M)!}{(2J)!} \left(\frac{(2J-1)!}{(J+M-1)!(J-M)!} - \frac{(2J-1)!}{(J+M)!(J-M-1)!} \right) &= \frac{M}{J}, \end{aligned} \tag{A3}$$

where standard combinatorics methods have been used to determine the number of permutations resulting in nonzero values of the scalar product. One can notice that the computations presented in Eq. (A3) are inconsistent with the result from Ref. [2], where the inverse expression $-M/J$ was reported. However, this discrepancy does not affect the probability of dephasing, which is equal to the square of the modulus of $\langle J,M,\sigma| 2\hat{s}_j^{(\sigma)z} |J,M,\sigma\rangle$. The result Eq. (A3)plays a key role in the following sections, where we compare individual terms of the GKLS equation as presented in Ref. [2] with the corrected formulation developed in the present contribution.

Over time, atoms in the collective Dicke state $|J,M,\sigma\rangle$ become decoupled. It manifests itself in the breakdown of the state describing the group of color centers. The initial state for one decoupled atom is a superposition of the state of such an atom in either the excited $|e_\sigma\rangle$ or ground state $|g_\sigma\rangle$ with the Dicke state with the J number reduced by half and the M number increased or decreased. The M number for the Dicke state in said superposition is reduced by $1/2$ when an atom in the excited state has been decoupled, and increases by $1/2$ when such a single emitter was in the ground state. The dephasing process and the described superposition of states are presented in Eq. (A4). An atom that has been decoupled to its own independent subspace, which is no longer a collective emitter space, will emit (or not when in the ground state) according to the standard Weisskopf-Wigner law. The dephasing effect can be shown in the following mathematical way:

$$|J,M,\sigma\rangle \xrightarrow{2\hat{s}_j^{(\sigma)z}} \sqrt{\frac{J+M}{2J}} |e_\sigma\rangle_j \otimes \left| J-\frac{1}{2}, M-\frac{1}{2}, \sigma \right\rangle - \sqrt{\frac{J-M}{2J}} |g_\sigma\rangle_j \otimes \left| J-\frac{1}{2}, M+\frac{1}{2}, \sigma \right\rangle, \tag{A4}$$

where $|e_\sigma\rangle$ denotes the independent excited state for one color center in the domain σ.

Let us start to analyze the differences in the individual terms of the fluorescence equations presented in Supplementary Table 1. First, we note that in the case of term I, taken from Eq. (3) in the main text, there

is a difference in the sign (+ in Model A and − in Model B). This inconsistency does not appear to arise from differences in interpretation, but rather from the mathematical foundation, which are expected to be consistent across both models. It relates to the derivation of the fluorescence formula for a fixed number of emitters, a procedure well documented in the literature, e.g., in Refs. [5, 6].

In Ref. [5], the formula for the coefficient γ describing the transition from the state $|J,M,\sigma\rangle$ with quantum number M to the state with $M-1$ is given in Eq. 2.17. A similar expression appears in Ref. [6] as Eq. 1.2.17. The latter takes the form:

$$\gamma_{M,M-1} = \gamma\left(\frac{1}{2}N + M\right)\left(\frac{1}{2}N - M + 1\right), \tag{A5}$$

and by setting $N = 2J$, we obtain:

$$\gamma_{M,M-1} = \gamma\,(J+M)\,(J-M+1)\,. \tag{A6}$$

It is worth noting that Eq. (1) contains this expression correctly in its first line, where two terms appear: one corresponds to the transition from the state with $M+1$ to M, and the other from M to $M-1$. If we group the constants differently, we can rewrite the transition rate in an alternative but equivalent form:

$$\gamma_{M,M-1} = \gamma(J+M)(J-M+1) = \gamma((J^2+J)-(M^2-M)) = \gamma(J(J+1)-M(M-1)). \tag{A7}$$

The expression Eq. (A7) should serve as the appropriate structure for the terms in the third line of Eq. (1), except that here the relevant decay constant should be γ_{ISC}, since we are referring to the ISC transitions. Therefore, based on the derivation in Ref. [6], it follows that terms F and G should include additional factors in parentheses, as shown in Eq. (4) in the main text for Model B.

Moreover, according to Eq. 1.2.19a in Ref. [6], which refers to fluorescence, the rate equation involves a sum over products of $\gamma_{M,M-1}$ and P_M. Finally, Eq. (A7) also supports the correction we propose in term I identified in Eq. (3), where the sign in Model B is reversed relative to the version in Model A.

It is also worth noting that there is no term accounting for fluorescence from independent emitters in the expressions found in Ref. [6]. This is expected, as the book discusses Dicke superradiance in the absence of dephasing, which is the mechanism responsible for generating independently radiating atoms.

Furthermore, we note a disparity in terms F and G in Supplementary Table 1, where the discrepancy arises from the absence of one component of the product within two brackets. If this term serves as a phenomenological analogy to the solution of the GKLS equation for dephasing effects and non-radiative transitions, then substituting the appropriate structures of the coefficients denoted as J and M yields results consistent with Model B.

Here, the primary discrepancy between the equations in Models A and B lies in the majority of the probability coefficients that modify the γ constants accordingly. This discrepancy raises an intriguing issue, as the differences in these coefficients carry distinct interpretations.

In Model B, we assume that the dephasing constants or transitions are multiplied by the probability of dephasing from a given collective space to a smaller one, or alternatively, for non-radiative transitions within the full collective state. Additionally, this multiplication should account for the number of populations in this collective state and the number of states from which such transitions can occur. Consequently, each phenomenological component corresponds to the system dynamics for the fundamental component with the constant γ, which directly results from the GKLS equation without any phenomenological components. However, each component possesses different constant values and probabilities due to its inherent nature.

We hypothesize that the authors of the paper under critique might have utilized not the transition probabilities, but rather the probabilities of remaining in a given state. Consequently, the differences in the

components describing the probability of transition arise. Let us note that our line of reasoning was as follows. Operator $2\hat{s}_j^{(\sigma)z}$ is Hermitian. Hence we can consider the element $\langle J,M,\sigma|\,2\hat{s}_j^{(\sigma)z}\,|J,M,\sigma\rangle = M/J$ as $\left[\langle J,M,\sigma|\,2\hat{s}_j^{(\sigma)z}\right]|J,M,\sigma\rangle$, where:

$$\begin{aligned}\left[\langle J,M,\sigma|\,2\hat{s}_j^{(\sigma)z}\right]^{\dagger} &= 2\hat{s}_j^{(\sigma)z}\,|J,M,\sigma\rangle = \\ &= \sqrt{\frac{J+M}{2J}}\,|e_\sigma\rangle_j \otimes \left|J-\frac{1}{2},M-\frac{1}{2},\sigma\right\rangle - \sqrt{\frac{J-M}{2J}}\,|g_\sigma\rangle_j \otimes \left|J-\frac{1}{2},M+\frac{1}{2},\sigma\right\rangle,\end{aligned} \tag{A8}$$

is the state after dephasing. In this context, we can interpret $\langle J,M,\sigma|\,2\hat{s}_j^{(\sigma)z}\,|J,M,\sigma\rangle$ as a projection of the collective state onto the dephased state. Accordingly, the result from Eq. (A3) provides the probability amplitude for the transition from the collective state $|J,M,\sigma\rangle$ to the dephased state $2\hat{s}_j^{(\sigma)z}\,|J,M,\sigma\rangle$. By taking the square of the modulus of this amplitude, we obtain the probability that dephasing will occur. This probability is the quantity that should appear in the master equation governing the fluorescence dynamics. Therefore, the correct expression is $\left|\frac{M}{J}\right|^2$, and not $\left(1-\left|\frac{M}{J}\right|^2\right)$ as used in Eq. (1). This correction directly accounts for the changes we have introduced in the C, E, and H terms in Supplementary Table 1, which reflect the consistent and physically justified structure of the revised model.

Let us now examine the consequences of the result obtained in Eq. (A3), particularly the fact that we do not account for the probability of the reverse process. The squared modulus of the matrix element, $\left|\langle J,M,\sigma|\,2\hat{s}_j^{(\sigma)z}\,|J,M,\sigma\rangle\right|^2 = \left|\frac{M}{J}\right|^2$, represents the probability that the state $|J,M,\sigma\rangle$ undergoes dephasing. This follows from interpreting the matrix element as a projection of the collective state onto its dephased counterpart, $2\hat{s}_j^{(\sigma)z}\,|J,M,\sigma\rangle$.

It means that for $M=0$, the dephasing probability is zero. This implies that the population $P_{J,M}^{(\sigma)}$ of the state $|J,M,\sigma\rangle$ cannot decay due to dephasing alone when $M=0$. For such states, the population $P_{J,M}^{(\sigma)}$ can be reduced only through other mechanisms–namely, ISC transitions associated with γ_{ISC}^{σ} and radiative decay characterized by the rate constant γ.

If we isolate the contribution from dephasing in Eq. (4), the population dynamics due to this mechanism alone takes the form:

$$\frac{d}{dt}P_{J,M}^{(\sigma)} = -\gamma_d^{\sigma}\left[2J\left|\frac{M}{J}\right|^2 P_{J,M}^{(\sigma)}(t) - 2\Big(J+\frac{1}{2}\Big)\left|\frac{M+\frac{1}{2}}{J+\frac{1}{2}}\right|^2 P_{J+\frac{1}{2},M+\frac{1}{2}}^{(\sigma)}(t)\right]. \tag{A9}$$

In particular, for $M=0$, this yields a positive contribution to the population:

$$\frac{d}{dt}P_{J,0}^{(\sigma)} = 2\gamma_d^{\sigma}\Big(J+\frac{1}{2}\Big)\left|\frac{\frac{1}{2}}{J+\frac{1}{2}}\right|^2 P_{J+\frac{1}{2},\frac{1}{2}}^{(\sigma)}(t). \tag{A10}$$

This result is not surprising: since dephasing does not deplete the population $P_{J,0}^{(\sigma)}$, it can only increase due to inflow from higher J states. Of course, this is just one component of the full population dynamics. When the complete models as in Eq. (4) is considered, the state with $M=0$ will also lose population via intersystem crossing and radiative decay.

Now let us refer to terms A, B, and D in Supplementary Table 1. In our opinion, these differences stem from a misplacement of the factor $2J$, which in Eq. (1) is applied in a way that it multiplies all components

parameter	$N=2$	$N=7$	$N=10$
$p_{\sigma=0}$	0.56	0.51	0.50
γ [2π MHz]	2.5	4.8	3.3
$\gamma_d^{\sigma=\pm1}$ [2π MHz]	270	260	420
$\gamma_d^{\sigma=0}$ [2π MHz]	27	20	39
$\gamma_{ISC}^{\sigma=\pm1}$ [2π MHz]	9.4		
$\gamma_{ISC}^{\sigma=0}$ [2π MHz]	1.8		

Supplementary Table 2. In the table, we present all parameters employed in the simulation of the variants under consideration. All values are consistent with those reported in Ref. [1]

inside the dephasing bracket. However, this factor should multiply only the first term within the bracket, as it corresponds to the decay of $P_{J,M}$. In Ref. [2], it incorrectly multiplies both terms, including the one involving $P_{J+\frac{1}{2},M+\frac{1}{2}}$, which already carries its own prefactor of $2(J+\frac{1}{2})$. This inconsistency likely results from treating both terms as if they referred to the same J, when in fact they belong to different subspaces.

Moreover, we argue that the plus sign inside the bracket in term D should in fact be a minus sign. This follows both from the modeling logic in the first line of Eq. (1), which serves as the basis for the phenomenological terms, and from a quick consistency check. If we retain the expression as given in the second line of Eq. (1) and multiply out the bracket, we obtain a following expression that supposedly governs the dephasing:

$$-2J\gamma_d^{\sigma}\left(1-\left|\frac{M}{J}\right|^2\right)P_{J,M}^{(\sigma)}(t)-2J\gamma_d^{\sigma}\cdot 2\left(J+\frac{1}{2}\right)\left(1-\left|\frac{M+\frac{1}{2}}{J+\frac{1}{2}}\right|\right)P_{J+\frac{1}{2},M+\frac{1}{2}}^{(\sigma)}(t). \tag{A11}$$

This would imply that the population $P_{J,M}$ decreases twofold due to dephasing: once directly through the dephasing of $|J,M,\sigma\rangle$, and again through the dephasing of $\left|J+\frac{1}{2},M+\frac{1}{2},\sigma\right\rangle$. The latter is incorrect—the correct effect of dephasing of the higher-J state should be to increase the population of $P_{J,M}$, which requires a positive sign in the second term. Therefore, we conclude that the use of the sign in the element D of Eq. (1) is a mistake and should be corrected to ensure physically consistent behavior.

Appendix B: Differences in simulations results and non-physical effects in Model A

We employ specific parameters in our simulations, all of which correspond to those used in Ref. [1]. This choice ensures a fair comparison between Models A and B under consistent conditions. Supplementary Table 2 lists the parameter values for 2, 7, and 10 color centers, as simulations involving these quantities are featured throughout the paper. Our approach involves simulating the original equations from Model A and comparing them with the modified equations from Model B, as presented in Supplementary Table 1. This comparative analysis serves as direct evidence indicating that Model B corrects the unphysical behavior of Model A.

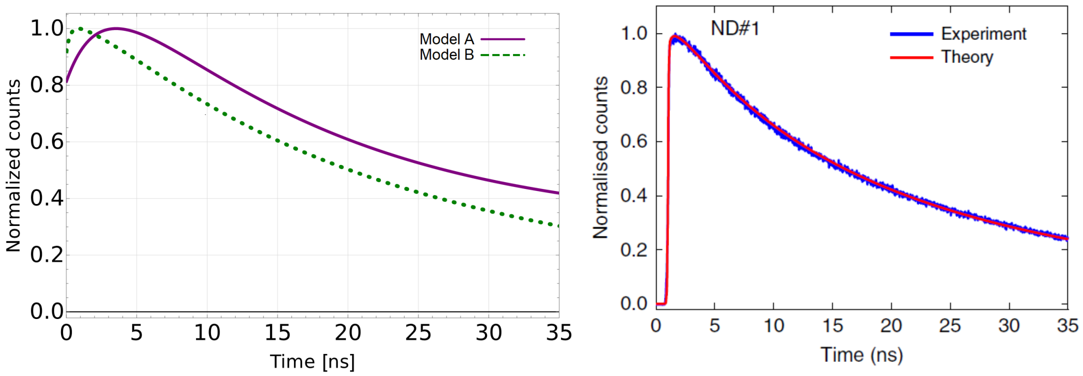

Supplementary Figure 1. In Figure on the left, a comparison of the fluorescence intensity between Model A (purple solid line) and Model B (green dashed line) for 2 NV centers is presented. Figure on the right is taken from Ref. [1] and presents the simulation results of Model A against experimental data.

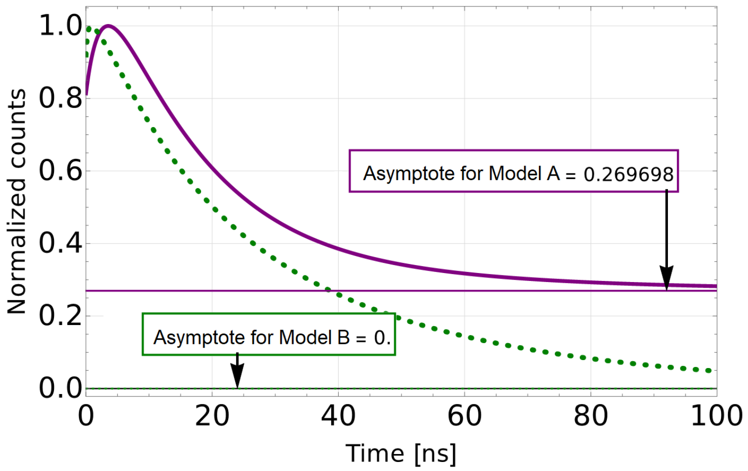

Supplementary Figure 2. The results of two simulation variants of fluorescence intensity of timespan from 0 to 100 ns, for 2 NV centers. The purple solid line represents Model A, while the green dashed line is based on Model B. The asymptotic values for both models are provided.

1. $N = 2$

We begin by simulating the fluorescence process from the equations of Models A and B for a time range from 0 to 35 ns for $N = 2$ color centers. Supplementary Figure 1 presents the normalized counts obtained through our simulations alongside those obtained by the authors of the referenced paper [1].

The time limit of 35 ns provides a convenient scale for comparison. Notably, we observe a non-zero initial fluorescence in our results, a phenomenon that is consistent with expectations as it typically does not originate from zero. While this effect is evident in plot from [1], it may be attributed to experimental data

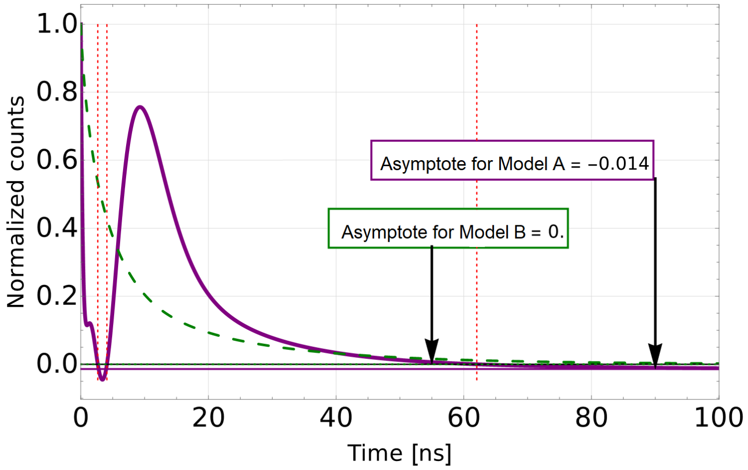

Supplementary Figure 3. The results of two simulation variants of fluorescence intensity in time from 0 to 100 ns, for 7 NV centers. The purple solid line represents Model A, while the green dashed line comes from Model B. Red dashed vertical lines are drawn to indicate where the values of the solid graph change the sign. The asymptotic values calculated for both models are also provided.

fitting, accounting for laboratory-induced effects. Moreover, our results exhibit no significant deviation at the 35 ns.

Firstly, it is important to highlight that in Supplementary Figure 1, our simulations based on Model A yield slightly different results compared to those obtained through Model B. Though the disparity is currently minor, it may become more pronounced with a larger number of centers. Secondly, we observe notable deviations between the simulated results from Model A and both the experimental data and the reported simulation outcomes provided in Ref. [1], as depicted in Supplementary Figure 1 (plot on the right).

Subsequently, we extend our analysis to plot the simulated fluorescence graph for two color centers over a broader time scale ranging from 0 to 100 ns, as illustrated in Supplementary Figure 2. This extended time frame effectively captures long-term discrepancies between Models A and B. Notably, the chart also depicts the asymptotes calculated for both models. In our model, the fluorescence asymptote is 0, reflecting a typical physical outcome. Conversely, in the Model A, fluorescence persists even at infinity.

2. $N = 7$

For $N = 7$, the intensity plot Supplementary Figure 3 obtained from Model A exhibits a sharp decline followed by erratic fluctuations in intensity, culminating in an abrupt rise to a significant value before subsequently decreasing. Upon simulating this scenario for a duration of 100 ns and beyond, an unexpected outcome emerges – the photon counts register negative values. To emphasize these inconsistent effects, we have incorporated red vertical lines into the simulated charts, marking instances where the fluorescence value reaches 0. This visual aid facilitates the identification of instances where the charts indicate a change in the sign of fluorescence, which represents an unphysical characteristic. Assuming $N = 7$, we also computed asymptotic values for Models A and B. While Model A yields a negative asymptote, which is physically unrealistic, Model B proposed in this paper converges to a zero asymptote, aligning with expected physical behavior.

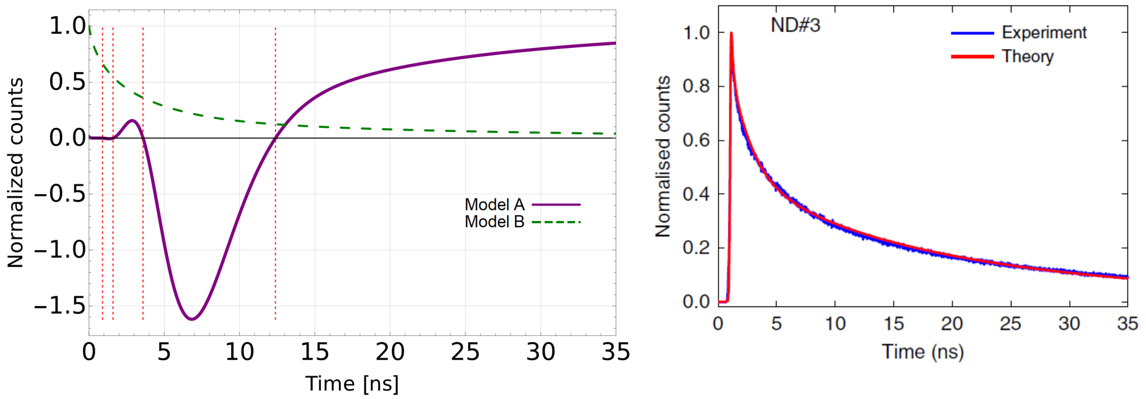

Supplementary Figure 4. In Figure on the left, a comparison of the fluorescence intensity between Model A (purple solid line) and Model B (green dashed line) for 10 NV centers is presented. Figure on the right is taken from Ref. [1] and presents their simulation results of Model A against experimental data.

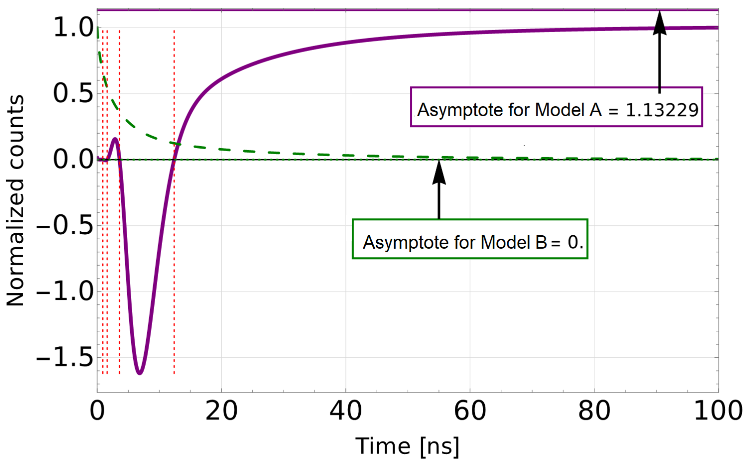

Supplementary Figure 5. The result of two simulation variants of fluorescence intensity in time from 0 to 100 ns, for 10 NV centers. The purple solid line represents Model A, while the green dashed line is based on Model B. Red dashed vertical lines are drawn to indicate where the values of the solid graph change the sign. The asymptotic values calculated for both models are also provided.

3. $N = 10$

Supplementary Figure 4 shows the same considerations for $N = 10$ and the parameters taken from Supplementary Table 2. Again, our simulations based on Model A starkly contrast with those presented in the referenced paper [1]. Additionally, immediate detection of unphysical effects is evident. Initially, fluorescence nearly reaches zero for several nanoseconds, then sharply increases before declining to strongly negative photon count values. Subsequently, it returns to positive values and stabilizes at an asymptote representing maximum positive intensity. On the other hand, results for Model B do not feature any anomalies

(see the green dashed line in Supplementary Figure 4).

Supplementary Figure 5 displays a broader time interval, up to 100 ns, revealing that a constant fluorescence persists even without any external excitation, which cannot be supported by any physical arguments. We observe that within Model B, the fluorescence intensity correctly converges to the asymptotic zero value, indicating that fluorescence vanishes at infinity.

[1] Bradac, C., Johnsson, M., Breugel, M. et al. Room-temperature spontaneous superradiance from single diamond nanocrystals. *Nat. Commun.* **8**, 1205 (2017).

[2] Bradac, C., Johnsson, M., Breugel, M. et al. Supplementary Information to Room-temperature spontaneous superradiance from single diamond nanocrystals. *Nat. Commun.* **8**, 1205 (2017).

[3] Czerwinski A. Dynamics of Open Quantum Systems–Markovian Semigroups and Beyond. *Symmetry* **14**, 1752 (2022).

[4] Borkowski, J. J., Czerwinski, A. & Kolenderski, P. Simulation Code for Superradiant Fluorescence from NV Centers in Diamonds. ResearchGate (2025). https://doi.org/10.13140/RG.2.2.12161.11364

[5] Gross, M. & Haroche, S. Superradiance: An essay on the theory of collective spontaneous emission. *Phys. Rep.* **93**, 301–396 (1982).

[6] Benedict, M., Ermolaev, A., Malyshev, V. et al. Super-radiance: Multiatomic Coherent Emission (Taylor & Francis Group, New York, 1996).
